# Selective Adsorption of Hazardous Substances from Wastewater by Hierarchical Oxide Composites: A Review

**DOI:** 10.3390/toxics12070447

**Published:** 2024-06-21

**Authors:** Wenjun Tu, Weiquan Cai

**Affiliations:** Guangzhou Higher Education Mega Center, School of Chemistry and Chemical Engineering, Guangzhou University, 230 Wai Huan Xi Road, Guangzhou 510006, China; 19972083102@163.com

**Keywords:** hierarchical oxide composites, preparation method, hazardous pollutants, selective adsorption, selective adsorption mechanism

## Abstract

Large volumes of wastewater containing toxic contaminants (e.g., heavy metal ions, organic dyes, etc.) are produced from industrial processes including electroplating, mining, petroleum exploitation, metal smelting, etc., and proper treatment prior to their discharge is mandatory in order to alleviate the impacts on aquatic ecosystems. Adsorption is one of the most effective and practical methods for removing toxic substances from wastewater due to its simplicity, flexibility, and economics. Recently, hierarchical oxide composites with diverse morphologies at the micro/nanometer scale, and the combination advantages of oxides and composite components have been received wide concern in the field of adsorption due to their multi-level structures, easy functionalization characteristic resulting in their large transport passages, high surface areas, full exposure of active sites, and good stability. This review summarizes the recent progress on their typical preparation methods, mainly including the hydrothermal/solvothermal method, coprecipitation method, template method, polymerization method, etc., in the field of selective adsorption and competitive adsorption of hazardous substances from wastewater. Their formation processes and different selective adsorption mechanisms, mainly including molecular/ion imprinting technology, surface charge effect, hard-soft acid-base theory, synergistic effect, and special functionalization, were critically reviewed. The key to hierarchical oxide composites research in the future is the development of facile, repeatable, efficient, and scale preparation methods and their dynamic adsorption with excellent cyclic regeneration adsorption performance instead of static adsorption for actual wastewater. This review is beneficial to broaden a new horizon for rational design and preparation of hierarchical oxide materials with selective adsorption of hazardous substances for wastewater treatment.

## 1. Introduction

With industrial development, wastewater treatment has become one of the most serious environmental problems. The major pollutants, including metal ions, dyes,and other toxic organics, in the effluents seriously harm biodiversity, ecosystem functions, and aquatic systems. Some methods, including ion exchange, chemical precipitation, adsorption, membrane separation, and electrochemistry treatment, are applied for wastewater treatment. Among them, adsorption has aroused widespread concern because of its convenient and flexible operation, high efficiency, and friendly and regenerative characteristics. Furthermore, some pollutants are valuable and can be reused after separation from the pollution system. Therefore, it is necessary and urgent to rationally design and prepare novel materials with special selectivity towards specific pollutants for different situations.

In the past years, many hierarchical oxides with multi-level structures and diverse morphologies resulting in improved physicochemical and surface properties and potential applications including photocatalysis [1], ion detection [2], membrane separation [3], and adsorption [4] have been reported. The construction of their structures is usually considered to be a process of self-assembly from a low-dimensional structure to a high-dimensional structure. Commonly, hierarchical oxide has the advantages of a high surface area, ease of modification, special application structure, and various morphologies. Especially, the well-structured hierarchical structure with an interconnected porous network facilitates the movement of pollutants to the exposing active sites located on the pore walls, resulting in better diffusion and adsorption process. Therefore, with rational design, preparation, and modification of functional groups, hierarchical oxide composites show great potential for selective adsorption of specific pollutants from wastewater.

Applications of hierarchically structured metal oxides, including cobalt oxide, iron oxide, and ceria, for the removal of As(V) and Cr(VI) ions in water [5], metal oxide heterostructures for arsenic removal from contaminated water [6], MgO with micro-nanostructures and composites of nano-MgO-based materials focusing on their composition and application [7], and different hierarchical nanostructures of TiO_2_ for energy and environmental applications [8] have been concisely reviewed. Thereafter, rationally designing appropriate hierarchical oxide composites for effective removal of hazardous pollutants has been the top strategy for wastewater treatment. Herein, recent research progress on their development and selective adsorption of hazardous substances from wastewater, together with their selective adsorption mechanisms, was presented to deepen their understanding and promote their applications in wastewater treatment. Especially, our focus is on the effects of their hierarchical structures with diverse morphologies instead of hierarchical porous structures with multi-level pores, including macro-, meso-, and micropores.

## 2. Preparation of Hierarchical Oxide Composites with Selective Adsorption Abilities

### 2.1. Hydrothermal/Solvothermal Method

**Figure 1:**
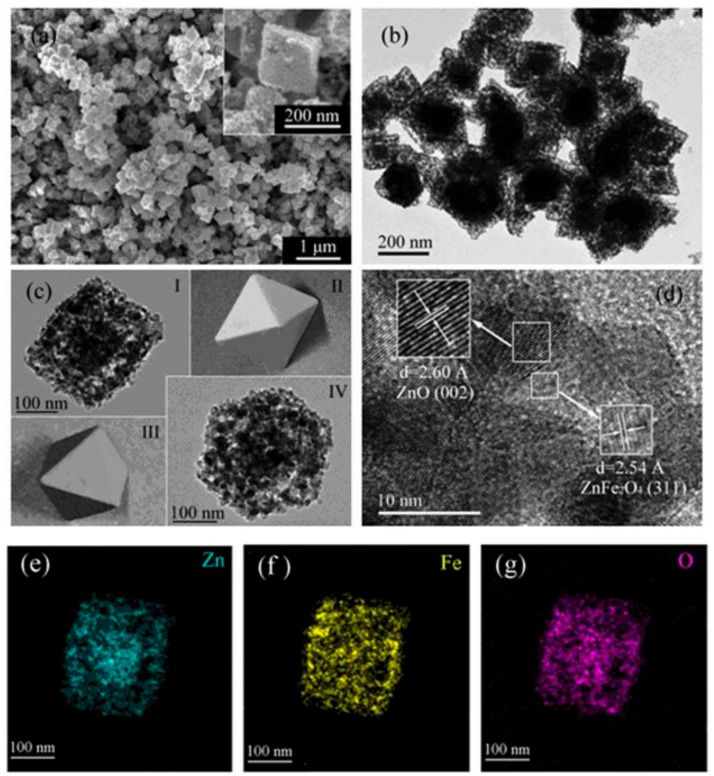
(**a**) FESEM (inset is a high solution image of a particle), (**b**,**c**) FETEM, (**d**) HRTEM images of the ZnO/ZnFe_2_O_4_ nanoparticles; EDS elemental mapping images to the corresponding area: (**e**) Zn, (**f**) Fe, and (**g**) O [11]. (In (**c**), images I and IV are the TEM scannograms from different perspectives of a same octahedral particle, photo-illustrated as images II and III, respectively).

The hydrothermal/solvothermal method is widely used to prepare hierarchical materials via dissolution and recrystallization processes in a sealed pressure vessel. It has the advantages of a relatively uniform distribution of particle sizes, poor particle aggregation, complete grain development, and easy control of the morphology in a specific direction. It is worth noting that polyols are usually used as solvents, such as ethylene glycol (EG) [9] and triethylene glycol [10], and under solvothermal conditions, the mixed water-polyol solvent plays a key role in controlling the self-assembly of oxide nanoparticles to form a hierarchical morphology. For example, Liu et al. [11] adopted a modified hydrothermal process and followed heat treatment to fabricate an octahedral ZnO/ZnFe_2_O_4_ composite using FeCl_3_·6H_2_O and ZnCl_2_ as the metal resources, CH_3_COONa as the assistant, and EG as the solvent. Figure 1 shows that the octahedron composite presents particle sizes of about 200 nm composed of three elements (Zn, Fe, and O) uniformly. The ZnO/ZnFe_2_O_4_ can adsorb malachite green (MG) with a maximum adsorption capacity as high as 4983.0 mg·g^−1^ from mixed dye solutions including methyl orange (MO) and rhodamine B (RhB) via ion exchange. Due to that, its pore volume and pore sizes can be adjusted by different heat treatments. Hierarchical rod-like CuO with a remarkable affinity for hazardous dichromate adsorption properties was also hydrothermally synthesized, and its inherent self-assemble structure and high surface area allowed the creation of effective adsorption sites. However, it is interesting that rod-shaped CuO nanomaterials could not be obtained in the absence of EG [12]. EG was also used as a solvent in our lab to prepare Fe_3_O_4_ microspheres with an average diameter of about 500 nm. After modification with chitosan, the composite showed more than 90% removal efficiency for Cr(VI) in the presence of various cations, including Cd^2+^, Cu^2+^, Zn^2+^, and Ni^2+^, or anions, including Cl^−^, C_2_O_4_^2−^, SO_4_^2−^, and HPO_4_^2−^ [13]. During the hydrothermal process, the growth of nanoparticles was achieved by the condensation of hydroxyl groups in alcohols, resulting in the local formation of trace water.

Hydrothermal time is an important factor that significantly affects the formation of hierarchical structures. Wu et al. [14] used a similar method to prepare porous α-MoO_3_ for selective removal of Pb^2+^ in a solution containing Cu^2+^, Zn^2+^, Cr^3+,^ and Cd^2+^ through the formation of lead molybdate by MoO_3_ and Pb^2+^ as follows:

MnO_3_ + H_2_O +Pb^2+^ → PbMnO_4_ + 2H^+^ (1)

The SEM with different times shows that the α-MoO_3_ nucleuses can gradually aggregate to form flower-like microspheres with diameters of 2.5–4.5 μm composed of massive nanobelts. Then, they grew on capillaries to obtain hand-like nanosheet arrays along the preferential orientation.

Some researchers used hierarchical oxides of rare earth elements, including La-, Ce-, to remove pollutants. For example, Chen et al. [15] prepared a three-dimensional (3D) graphene-La_2_O_3_ composite composed of graphene and La_2_O_3_ particles, which were prepared in advance and then heated together. La- could bind to graphene through La-O, and the composite achieved 100% removal efficiency for phosphate through La-O-P in the presence of Cl^−^, SO_4_^2−^, and NO_3_^−^. Rotzetter et al. [16] also proved that La_2_O_3_ could adsorb phosphate as follows:

La_2_O_3_ + 2HPO_4_^2−^ + H_2_O → 2LaPO_4_ + 4OH^−^ (2)

Likewise, Sun et al. [17] used La- to synthesize lanthanum molybdenum oxide with an average diameter of about 270 nm, and it showed almost removal efficiency of 100% for various mixed dyes with multi-sulfonic groups. Because La- center lacks electrons, it can attract electron-rich groups such as sulfonic groups. Furthermore, Tong et al. [18] reported a cerium oxide doped by two-dimensional (2D) molybdenum disulfide nanosheets. CeO_2_ nanoparticles (3–5 nm) could grow on 2D MoS_2_, and the maximum adsorption capacity of MoS_2_/CeO_2_ nanohybrids for Pb^2+^ is 333 mg·g^−1^ at a pH value of 2.0. It also showed an amazing selective adsorption for Pb^2+^ from the coexisting ions of K^+^, Na^+^, Ca^2+^, Mg^2+^, Mn^2+^, Cr^3+^, Co^2+^, Ni^2+^, Zn^2+^, Cu^2+,^ and Cd^2+^ due to the coordination effect between S and Pb.

**Figure 2:**
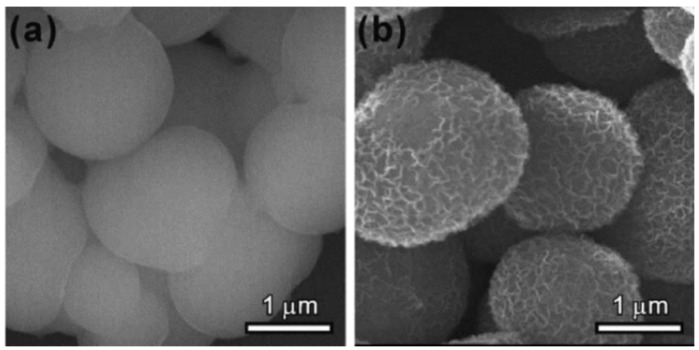
SEM images of the products at the early stage of the reaction: (**a**) 2 h, and (**b**) 6 h [20].

The development of TiO_2_-based composites for pollution removal has also attracted the attention of researchers. For example, Lee et al. [19] reported a hydrothermal preparation of flower-like TiO_2_-graphene oxide (GO). Nonmetallic oxides GO (100 nm in diameter) with oxygen-containing functional groups was used as a supporting matrix to induce the self-assembled growth of 3D flowerlike TiO_2_ nanoparticles. The GO-TiO_2_ showed the following adsorption order: Zn^2+^ > Cd^2+^ > Pb^2+^ due to ionic radius or electronegativity effects. Similarly, Xie et al. [20] designed a simple method to synthesize layered protonated titanate hierarchical microspheres with average diameters of 2 μm and an extremely high specific surface area of 450 m^2^·g^−1^, and the self-assembly process was achieved through aggregation, crystallization, and growth processes (Figure 2). This material shows specific selectivity for methylene blue (MB) compared to MO, cresol red, and RhB, and the two key factors affecting adsorption capability are the electrostatic interaction and steric structure of the organic dyes.

Using waste biomass or carbon as a dispersion carrier of metal oxides to prepare composites could reduce their agglomeration, further enhancing their adsorption performance via a synergistic effect. For example, durian shell fibers modified with Cu-Al bimetallic oxide (Cu-Al/DBF) with hierarchical structure were hydrothermally synthesized, and the Cu-Al/DBF was used to remove ammonia nitrogen from wastewater. Its maximum adsorption capacity was 18.04 mg·g^−1^ fitted from the Langmuir isotherm due to the synergistic effect of biomass carbon, alumina, and copper oxide [21]. Furthermore, the magnetic Fe_3_O_4_@C hollow microspheres obtained by a solvothermal method coupled with an annealing strategy showed an adsorption capacity of 197.2 mg·g^−1^ for Cr(VI). Especially, the magnetic Fe_3_O_4_ core can provide easily separated characteristics by magnetic separation, and the carbon layers can effectively prevent the aggregation of the magnetic Fe_3_O_4_ nanoparticles [22].

Deep eutectic solvents (DESs), as a type of ionic liquid analog, are regarded as an alternative to conventional solvents in the fields of material chemistry. Li et al. [23] reported uniformly distributed MgO microcubes based on DES consisting of MgCl_2_·6H_2_O and urea with a molar ratio of 1:2 via a solvothermal method and the following calcination process. Their adsorption capacities for anionic dyes Congo red (CR), Amaranth, and Indigo carmine were 666.7, 43.74, and 54.32 mg·g^−1^, respectively, at 25 °C, relating to electrostatic attraction and hydrogen bonding.

### 2.2. Coprecipitation Method

Coprecipitation is a method of adding precipitant into a mixed metal salt solution to precipitate two or more cations together to form a precipitate. Its typical application is the preparation of magnetic Fe_3_O_4_, and this process achieves the transformation from zero-dimensional nanoparticles to 3D microspheres using Fe^2+^ and Fe^3+^ as the cores for forming 3D core-shell material. Wang et al. [24] designed a core-shell magnetic calcium silicate/GO; the average diameter of Fe_3_O_4_ nanoparticles coated by calcium silicate was about 10 nm. The composite showed selective adsorption for acridine orange via electrostatic, hydrophobic, and π-π interactions. Zavareh et al. [25] also reported a Cu-chitosan/Fe_3_O_4_ nanocomposite to selectively adsorb phosphate in the presence of chloride, nitrate, and sulfate, whose concentrations are 10 times higher than phosphate due to complex formation between Cu(II) and phosphate; the mean size of magnetite nanoparticles was between 20 and 30 nm.

This method can be extended to prepare other composite metal oxides. For example, Rahman et al. [26] synthesized Ag_2_O_3_-ZnO nanocones for selective Co(II) adsorption under the interference of Cd(II), Co(II), Cr(III), Cu(II), Fe(III), Ni(II), and Zn(II). The growth mechanism of nanocones could be described as a process of self-assembled nucleation and subsequent aggregation. The final product shows a 3D nanocone composed of nanoparticles, and the mean length and cross-section (center) of the nanocones were approximately 1.42 mm and 0.53 mm, respectively. Similarly, Marwani et al. [27] prepared a CdO coordinated Fe_2_O_3_ composite of aggregated nanofiber with an average width size of 70 nm using cadmium and ferric salts as metal resources and NaOH as precipitant, respectively. The selective research indicated that Pb(II) showed the highest distribution coefficient (*K_d_*) value of 1.21 × 10^5^ mL·g^−1^ among the coexisting cations, including Cd(II), Co(II), Cu(II), Cr(III), Cr(VI), Fe(III), Ni(II), and Zn(II). As another example, hierarchical Ti_3_C_2_@FeOOH nanocomposites for antimony-contaminated wastewater treatment were developed via in-situ anchored FeOOH into the interlamination of alkali-treated Ti_3_C_2_ nanosheets, and they showed excellent adsorption capacities for both Sb(V) and Sb(III), with the maximum adsorption capacities of 93.22 and 111.50 mg·g^−1^, respectively, based on isotherm analysis. They also showed superior selectivity, especially for Sb(III), irrelevant to foreign anions [28]. A flower-like AlOOH/AlFe intermetallic composite with a high adsorption rate and adsorption capacity for As(V) removal up to 200 mg·g^−1^ was prepared via direct precipitation of a bimetallic Al/Fe nanopowder with water at 60 °C, indicating that this micro/nanostructure with a specific surface area of 247.1 m^2^·g^−1^ facilitated deliverability [29].

**Figure 3:**
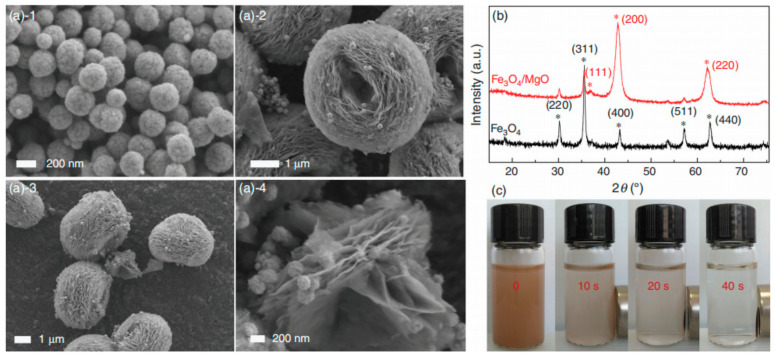
SEM images of Fe_3_O_4_ (**(a)-1**), Fe_3_O_4_/MgO (**(a)-2**–**(a)-4**), XRPD patterns of Fe_3_O_4_, Fe_3_O_4_/MgO (**b**) and the performance of magnetic separation (**c**) [31].

Hierarchical MgO is also being shown applications for removing various pollutants. For example, an EG-assisted precipitation route was developed to prepare 3D flower-like MgO samples at room temperature. The Mg^2+^ coordinates the -OH groups of EG to form an alkoxide coordination complex in a basic medium with different divalent cations to form sheets arranged in an ordered way to construct microspheres. The MgO showed the highest adsorption capacity of 574.71 mg·g^−1^ for phosphate [30]. Then, a 3D nest-like porous magnetic MgO hybrid (Fe_3_O_4_/MgO) with a specific surface area of 135.2 m^2^·g^−1^, uniform mesochannels of 5–35 nm, and easily separated characteristics (Figure 3) was controllably synthesized based on a seed-induced precipitation process containing a suspension of Fe_3_O_4_ nanoparticles with an average diameter of 200 nm and following calcination of the precursor. The Fe_3_O_4_/MgO had a good removal performance for simultaneous removal of 12 polycyclic aromatic hydrocarbons (PAHs) and Cd^2+^ with fast adsorption (~0.25 h) and high removal efficiencies (>70% for PAHs and >80% for Cd^2+^, respectively) [31].

### 2.3. Template Method

**Figure 4:**
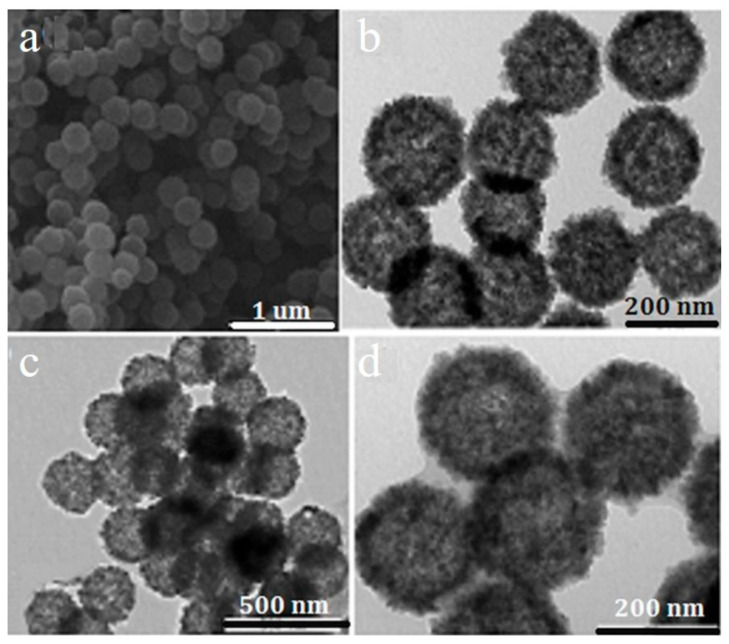
SEM (**a**) and TEM (**b**) images of hollow Fe_3_O_4_ precursor, and TEM (**c**,**d**) images of the as-prepared hollow porous magnetic Fe_3_O_4_@NH_2_-MIL-101(Fe) [32].

Template synthesis is a process in which materials with a nanostructure, unique morphology, and low cost are used as templates, and related materials are deposited into their holes or surfaces by chemical or physical methods. Then, the template is removed to obtain the desired hierarchical materials. Templates include soft templates and hard templates. Surfactants such as polyvinylpyrrolidone (PVP), cetyl trimethyl ammonium bromide (CTAB), polyethylene oxide-polypropylene oxide-polyethylene oxide (P123), etc. are used as soft templates to influence the morphologies and pore structures of the metal oxides. Li et al. [32] reported a PVP-assisted preparation method of hollow Fe_3_O_4_ with a mean diameter of 300 nm. Firstly, a hollow Fe_3_O_4_ precursor (Figure 4a,b) was prepared using FeCl_3_·6H_2_O assisted by sodium citrate and urea in deionized water; then, PVP was added following a hydrothermal process. Then, the PVP was removed by a 2-aminoterephthalic acid solution of ethanol to obtain hollow Fe_3_O_4_. Figure 4c depicts Fe_3_O_4_ with NH_2_-MIL-101(Fe) doped with inner hollow structures, while Figure 4d shows the layers belonging to NH_2_-MIL-101(Fe). The as-prepared adsorbent showed selective adsorption for phosphates in the presence of Cl^−^, F^−^, Br^−^, NO_3_^−^, and SO_4_^2−^.

Cai et al. [33] designed hollow C@SiO_2_ nanoparticles using CTAB as the template. Their mean sizes were approximately 500 nm, while the sizes of the core and the shell were 450 nm and 50 nm, respectively. CTAB was removed by ethanol and HCl solutions, resulting in a porous structure with a high specific surface of 150.57 m^2^·g^−1^. The adsorption capacity of C@SiO_2_ nanoparticles for Cr(VI) was 90.53 mg·g^−1^ in the presence of Cu(II), Cd(II), Zn(II), and Ni(II), which just declined 10% compared with no interfering cations. Furthermore, anions like Cl^−^ and NO_3_^−^ showed no influence on Cr(VI) adsorption, while SO_4_^2−^ and HPO_4_^2−^ could slightly affect it due to the similar hydration degree with HCrO_4_^−^. It was found that adding an appropriate amount of CTAB can change the morphology, pore properties of hierarchical MgO and its adsorption behavior for phosphate in a CTAB-assisted solvothermal route. Especially, the gardenia flower-like MgO with the highest specific surface area of 336.54 m^2^·g^−1^ and a total pore volume of 0.843 cm^3^·g^−1^ showed the highest adsorption capacity of 348.32 mg·g^−1^ for phosphate with a short equilibrium time of 4 h [34].

Hierarchical structures also include mesoporous materials, which possess several levels of structure via soft template modification. For example, Sarafraz et al. [35] used P123 as the template to prepare phosphonic functional groups-modified mesoporous silica microspheres for uranium selective adsorption, and P123 was removed via a boiled mixture of methanol and HCl. Similarly, Yang et al. [36] used P123 to synthesize phosphoric mesoporous silica with ion-imprinting technology for selective removal of uranium. The above researches showed that the phosphorous group is beneficial for selectively capturing uranium.

**Figure 5:**
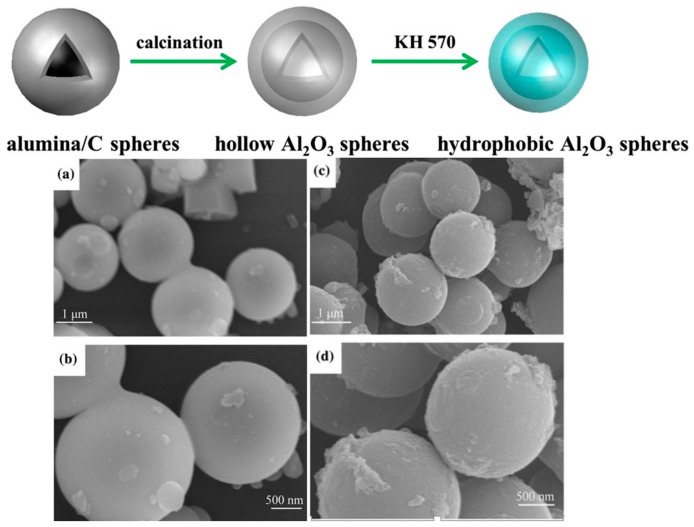
Schematic illustration for preparation and surface hydrophobic modification of Al_2_O_3_ spheres, SEM images of alumina/carbon spheres (**a**,**b**); and hollow Al_2_O_3_ microspheres (**c**,**d**) [37].

Unlike soft templates, hard templates, such as carbon, carbonate, and silica, can retain their morphologies in the sample. For example, Zhang et al. [37] prepared the porous Al_2_O_3_ microspheres/acrylic ester resin hybrids for selective absorption of oil and organic solvent via a microwave polymerization process by using Al_2_O_3_ spheres as modifiers. The Al_2_O_3_ microspheres with an average size of 2.0 μm were obtained using glucose as the carbon source of the carbon template from a combined hydrothermal and sintering processes, followed by surface modification with the silane coupling agent KH 570 (Figure 5) to enhance their hydrophobicity and reactivity. Pervaiz also reported a sacrificial carbonate template coupled with organic ligands and polymers for preparing cobalt iron oxide microspheres via a soft prototype route. They showed a high specific surface area of 786 m^2^·g^−1^ and a high magnetism of 63 emu·g^−1^, and were excellent adsorbents and catalysts for the oxidative-dissociation of RhB and 4-Nitrophenol from water at room temperature [38]. In another example, Zhang et al. [39] synthesized mesoporous δ-Bi_2_O_3_ using SBA-15 silicas prepared by different silicon sources as hard templates, which were later removed by a 2 M NaOH solution. The δ-Bi_2_O_3_ showed a selective adsorption capacity of 2.21 mmol·g^−1^ for I^−^ in the presence of Cl^−^.

### 2.4. Polymerization

**Figure 6:**
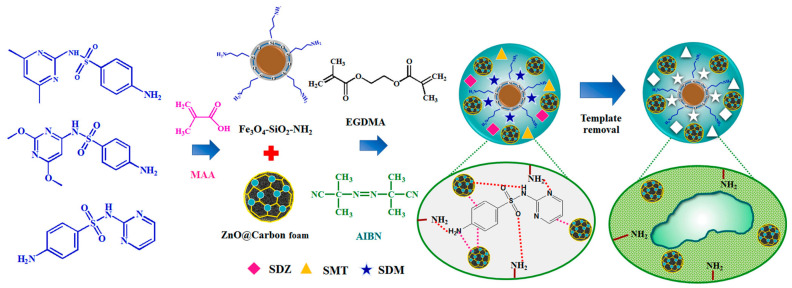
The preparation procedure of the proposed hierarchical nanocomposite ZnO@CF@Fe_3_O_4_-SiO_2_-NH_2_@MIP adsorbent for the extraction of sulfonamides [40].

Polymerization is usually used to combine polymers with metal oxides, and some core-shell structures are modified by polymers to obtain special functionality. For example, Kliangsuwan et al. reported a hierarchical composite which was incorporated a nanocomposite of zinc oxide and carbon foam embedded in a magnetic molecularly imprinted polymer (ZnO@CF@Fe_3_O_4_-SiO_2_-NH_2_@MIP) for extracting sulfonamides (Figure 6). The foam nanocomposite helped to improve the adsorption performance of sulfonamides; the molecularly imprinted polymer (MIP) provided highly specific recognition cavities for three sulfonamides, and the magnetic material enabled its simple and rapid separation after adsorption and desorption. This developed strategy determined sulfonamides in milk and water, with extraction recoveries between 84.3 and 96.2% [40]. In another example, Zhang et al. [41] prepared a Fe_3_O_4_/PANI/MnO_2_ core-shell hybrid with a diameter of 300 nm, and the coating thicknesses of polyaniline (PANI) and MnO_2_ shells could be controlled by determining the polymerization time and KMnO_4_ amount, respectively. It was found that the superior adsorption capacity of this hybrid for Cd(II), Zn(II), Pb(II) and Cu(II) was attributed to the synergetic effect between PANI and MnO_2_. Gu et al. [42] also designed an amino functionalized Fe_3_O_4_@SiO_2_ core-shell structure with a mean diameter of 320 nm by one-pot co-condensation. The Fe_3_O_4_@SiO_2_ modified by amino silane contained one N atom, and it showed the adsorption amount for multiple ions solution containing Cr(VI), Cu(II), Ni(II), Zn(II) and Cd(II) at the same time due to the abundant amino and hydroxyl groups of the adsorbent. Compared with that without interference ions, it is noteworthy that its adsorption capacity for Cr(VI) did not decrease. In addition, Hwang et al. [43] prepared a porous phenol resin containing lithium manganese oxide (LMO) through polycondensation and carbonization for selective adsorption of lithium. Poly(vinyl alcohol) (PVA) and hexamethylenetetramine were chosen as the stabilizer and the curing agent, respectively. Finally, the LMO microspheres with a rough surface were obtained. Hierarchical porous, magnetic Fe_3_O_4_@carbon nanofibers (Fe_3_O_4_@CNFs) based on polybenzoxazine precursors have been synthesized by a combination of electrospinning and in situ polymerization at 250 °C. The fibers with an average diameter of 130 nm were comprised of carbon fibers with embedded Fe_3_O_4_ nanocrystals and showed a high specific surface area of 1885 m^2^·g^−1^ and a pore volume of 2.3 cm^3^·g^−1^. The Fe_3_O_4_@CNFs showed efficient adsorption properties for organic dyes in water and excellent magnetic separation performance [44].

### 2.5. Other Preparation Methods

**Figure 7:**
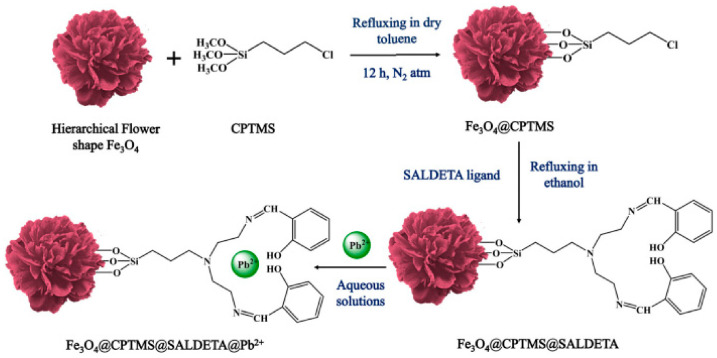
Schematic diagram of the surface modification of 3D hierarchical Fe_3_O_4_ structures and their adsorption for Pb^2+^ ions (CPTMS and SALDETA represent 3-chloropropyltrimethoxysilane and salicylaldehyde-diethylene triamine, respectively) [47].

Other preparation methods, mainly sol-gel, electrospinning, reflux, thermal decomposition, and chemical bath deposition, could also be used to prepare hierarchical oxide composites for pollutant removal via adsorption. Sol-gel method: hierarchical magnetic graphene oxide-titanate nanocomposites (MGO@TNs) with a high specific surface area of 193.4 m^2^·g^−1^ and magnetite nanoparticles anchored on them were prepared via a modified sol-gel and subsequent alkaline hydrothermal process. When 3 g·L^−1^ MGO@TNs was used for removing Pb(II) from stimulated realistic battery wastewater, safe discharge with a concentration lower than 0.05 mg·L^−1^ could be achieved due to ion exchange and surface complexation [45]. Electrospinning method: Min et al. [46] reported an electrospinning chitosan/Fe-Mn nanofibrous composite (Fe-Mn@CS NF) to remove trace As(III) from water, and its concentration decreased from 550 μg·L^−1^ to less than 1.2 μg·L^−1^ while using 0.5 g·L^−1^ Fe-Mn@CS NF. The presence of F^−^ or SO_4_^2−^ showed a negligible impact on As(III) removal, while PO_4_^3−^ impeded its adsorption via competing for adsorption sites. Reflux method: Solanki et al. [47] reported a 3D flower-like Fe_3_O_4_ architecture decorated with SALDETA moieties via refluxing the reaction mixture (Figure 7). This magnetical composite showed excellent adsorption capacity of 415.5 mg·g^−1^, faster kinetics of 8 min, rapid separation of 40 s, facile regeneration of 5 min, and good reusability of 5 runs for Pb^2+^ ions resulting from its hierarchical structure, immobilized functional groups, and chelation property. Electrostatic self-assembly method: to solve the disadvantages of easily distorted and aggregated into other uncontrolled morphologies for 2D lamellar-like graphene, resulting in a remarkable decline in performance, 3D macroporous reduced GO-Fe_3_O_4_ nanocomposites were synthesized via an electrostatic self-assembly method. They showed high adsorption capacities, rapid adsorption rates for Cr(VI), and easy magnetic separation for reusability. Interestingly, the Fe_3_O_4_ nanoparticles serve as stabilizers for separating graphene nanosheets from aggregation, while the graphene nanosheets favor hindering them from agglomeration and enabling their good distribution on the surface of graphene [48]. Thermal decomposition method: ferromagnetic 3D flower-like γ-Fe_2_O_3_ particles with an adsorption capacity of 102.7 mg·g^−1^ for CR were prepared by a simple direct thermal decomposition method using cheap and nontoxic ferric nitrate as an iron source and CTAB as a structure-directing agent. With increasing amounts of CTAB, the morphology of γ-Fe_2_O_3_ particles was transformed from 1D to 3D. Especially, the sample obtained by adding 15% CTAB showed a complete flower-like structure with smooth petals [49]. Chemical bath deposition method: hierarchical NiO hollow architectures (HPHAs) assembled from nanoflakes with a thickness of about 8 nm were synthesized via a one-pot facile chemical bath deposition method and the following calcination process. The HPHAs showed the maximum adsorption capacity of 490.2 mg·g^−1^ for CR from the Langmuir equation due to the synergistic effect of porous structure, large specific surface area, and the electrostatic attraction of NiO with CR molecules [50].

### 2.6. Non-Powder Adsorption Materials

All of the above adsorption materials are powders, and thus it is difficult to separate them after adsorption unless external forces, such as a magnetic field, are used. Non-powder materials such as membranes and aerogels with a 3D structure could solve this problem, and it is interesting that GO is popular to assist in their preparation. For example, Zhao et al. [51] synthesized a 3D aerogel based on GO modified by positively charged polyetherimide (PEI) via a sol-gel method. The GO/PEI aerogel with a tunable surface charge at different pH values was formed after freeze drying and was stable in acidic and basic aqueous solutions. It showed high adsorption capacities of 249.6 mg·g^−1^ for MB at pH 10.5 and 3331.0 mg·g^−1^ at pH 2.0 for anionic MO, respectively. Likewise, Rahmani et al. [52] prepared a N-doped reduced GO aerogel with a 3D inter-connected network via a hydrothermal method. This aerogel exhibited excellent selective adsorption performance for oil pollutants because of the coordination ability of N. Li et al. [53] designed a GO membrane functionalized with phenanthroline diamide (GO-PDA) through a modified sol-gel method.

Furthermore, SiO_2_ aerogel is a type of material that could be tailored in terms of its specific features and surface chemistry as an adsorbent for pollutant removal. For example, Lamy-Mendes et al. [54] synthesized methyltrimethoxysilane (MTMS)-based carbon nanostructures—silica aerogels via a two-step acidbase-catalyzed sol-gel process, and they can remove various organic compounds and drugs, and achieve adsorption capacities of 200 mg·g^−1^ for xylene and 170 mg·g^−1^ for toluene, respectively. The addition of co-precursors containing carbon nanomaterials and/or amine groups was a valuable tool to alter their properties, thus enhancing their adsorption performance. Zhang et al. [55] successfully fabricated flexible and hierarchical TiO_2_-SiO_2_ nanofibrous mats with superior adsorption efficiency and recyclable performance for methyl blue removal via sol-gel metnod and the following calcination process, and they could maintain integrated morphology after bending to curvature in which the radius is 0.6 mm, indicating that the brittleness of inorganic oxides was successfully overcome.

Membrane adsorption is an efficient and easily segregated method for pollutant removal. Park et al. [56] reported a chitosan-coated iron oxide nanocomposite immobilized hydrophilic poly(vinylidene) fluoride membrane (Chi@Fe_2_O_3_-PVDF) to remove Cr(VI) with adsorption capacities of 14.45 mg·g^−1^ in a batch system, and 14.10 mg·g^−1^ in a continuous in-flow system, respectively. Importantly, its removal efficiency was not changed significantly in the presence of competing ions, including Cl^−^, NO_3_^−^, SO_4_^2−^, and PO_4_^3−^. Our previous work [57] reported a γ-AlOOH/PVA membrane via a sol-gel method, and it presented a good adsorption capacity for Cr(VI), due to electrostatic interaction. The adsorption results of coexisting anions (HCO_3_^−^, HPO_4_^−^, C_2_O_4_^2−^, F^−^, Cl^−^, and SO_4_^2−^) indicated that the composite membrane showed a highly selective adsorption capacity for Cr(VI) while only HCO_3_^−^, SO_4_^2−^, and HPO_4_^2−^ could slightly cause interference due to the similar radii with HCrO_4_^−^. Moreover, both Sun et al. [58] and Tan et al. [59] used the sol-gel method with PVA as an adjuvant to form membranes. It seems that this sol-gel method, assisted by PVA, has the ability to make each component form a uniform 3D network structure. Hierarchical hybrid nanocomposite MgO@PES-PDA membranes were also constructed by coating MgO nanoparticles on the PES-PDA derived from co-deposition of polydopamine (PDA) and PEI under mild basic conditions, and such membranes showed a rapid capture and high destructive adsorption capacity for paraoxon toxin (up to 92% within 40 min). Especially the smaller MgO-coated PES-PDA membrane with relatively high MgO content showed the best destructive absorption ability [60].

The advantages and disadvantages of the different preparation methods for preparation of hierarchical oxides were compared in Table 1.

## 3. Selective Adsorption Mechanism

### 3.1. Molecular/Ion Imprinting Technology

**Figure 8:**
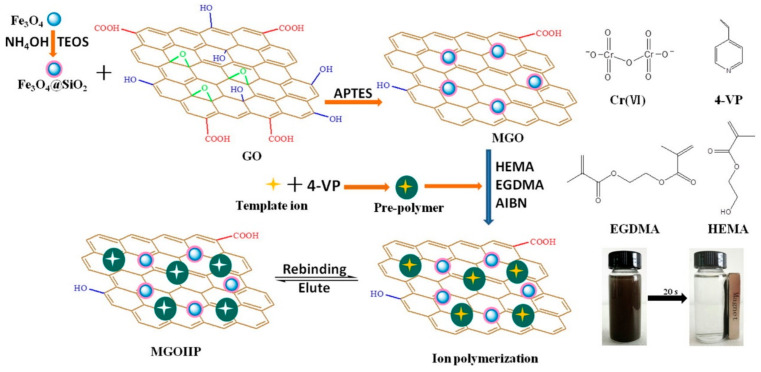
Schematic of synthesis route of Cr(VI) imprinted polymer (the meanings of the corresponding abbreviations are as follows: tetraethoxysilane (TEOS), 3-aminopropyltriethoxysilane (APTES), 4-vinyl pyridine (4-VP), 2-hydroxyethyl methacrylate (HEMA), ethylene glycol dimethacrylate (EGDMA), N,N-azoisobisbutyronitrile (AIBN), ion-imprinted polymer (MGOIIP)) [62].

Molecularly imprinted technology (MIT) was first used in biological detection. In the field of adsorption, ion imprinting technology (IIP), which is similar to MIP but uses ions as a template, has received extensive attention. MIT and IIP can generate specific binding sites for molecules and ions in the adsorbent with a non-biological strategy [61]. The adsorbent after removing the template showed extremely selective adsorption for target molecules or ions. Liang et al. [62] prepared a Cr(VI) ion-imprinted polymer using four organic compounds as chemical additives with different functions. Core-shell Fe_3_O_4_@SiO_2_ was introduced for easy separation, and GO can prevent Fe_3_O_4_@SiO_2_ from aggregation. The adsorbent reached adsorption equilibrium within 5 min for Cr(VI), and its adsorption capacity increased from 182.77 to 301.89 mg·g^−1^ compared with the sample without using IIP. Furthermore, its selectivity coefficient for Cr(VI) was high in the presence of Cu(II), Cd(II), Cr(III), Ni(II), SO_4_^2−^ and NO_3_^−^ indicating its enhanced selective adsorption performance for Cr(VI) by using IIP. Compared with cations, its adsorption was significantly affected by anions because of their similar charge and ionic radii with Cr(VI). Figure 8 showed that the template ions left identifiable sites in the material after washing by acidified thiourea solution; the memory effect enabled the adsorbent to adsorb Cr(VI) faster and selectively. In another example, Zhang et al. [63] used MIP to selectively adsorb 2-aminopyridine based on magnetic chitosan and β-cyclodextrin, and then a methanol-acetic acid solution was chosen to remove the template. Compared with the adsorbent obtained without template, the adsorption capacity of the adsorbent obtained with template increased three times, and it also showed a higher selectivity for 2-aminopyridine in the presence of its structural analogues or coexistent ions (Na^+^, K^+^, Mg^2+^, Ca^2+^, Cl^−^, and SO_4_^2−^) due to the recognition effect. Similar reports were also reported [64,65,66], and the key of MIP or IIP is to add the adsorbate to the adsorbent as the template. After removing the template, a highly selective adsorbent for this adsorbate can be obtained.

### 3.2. Electrostatic Interaction

#### 3.2.1. Surface Charge Effect

**Figure 9:**
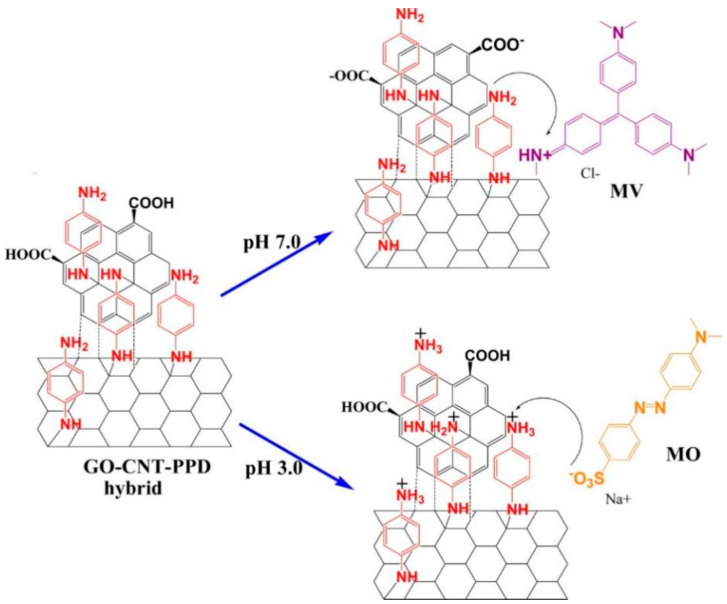
Effect of pH on adsorption of rGO−CNT−PPD hybrid for methyl violet (MV) and MO [70].

Because many pollutants are charged, selective adsorption can be achieved by choosing a material with appropriate opposite charges [67]. Chen et al. [68] designed a Fe_3_O_4_@NH_2_@PEI core/shell composite, and the average size of the Fe_3_O_4_@NH_2_ cores was 40 nm, while the average shell thickness was 40 nm. NH_2_- groups act as a bridge for PEI to be loaded on the magnetic core. Due to the fact that PEI is positively charged, it can selectively adsorb anionic dyes from a mixed solution of cationic and anionic dyes; the adsorption can reach equilibrium within 30 min. Likewise, Huang et al. [69] prepared a similar Fe_3_O_4_@Tb/AMP core-shell composite with a mean size of 10 nm. It showed similarly selective adsorption performance for anionic dyes because adenosine 5′-monophosphate monohydrate (AMP) was positively charged in the solution. Sarkar et al. [70] synthesized a more flexible amine-functionalized reduced graphene oxide-carbon nanotube hybrid (rGO-CNT-PPD) with an average width of 20 nm. As shown in Figure 9, rGO-CNT-PPD can selectively adsorb cationic dyes in an acidic environment, while it shows selective adsorption performance for anionic dyes in a neutral environment because of the change in surface charge.

#### 3.2.2. Hard-Soft-Acid-Base Theory

Hard-soft-acid-base theory (HSAB) claims that acids and bases are divided into hard and soft categories depending on their different properties. “Hard acid first binds to hard base, soft acid first binds to soft base” is one of the most important empirical rules to explain adsorption selectivity. Ashour et al. [71] prepared citric acid and l-cysteine-modified Fe_3_O_4_ microspheres (CA@Fe_3_O_4_ and Cys@Fe_3_O_4_, respectively) to selectively adsorb rare earth ions (RE^3+^). The coexistence cations (RE^3+^, Mg^2+^, Ca^2+^, and Ni^2+^) adsorption experiment showed that O and N in CA@Fe_3_O_4_ and Cys@Fe_3_O_4_ are hard Lewis base atoms, and thus they can selectively complex with RE^3+^ ions, which are hard Lewis acids. The authors used separation factor (SF) based on *K_d_* to describe selective ability, and further research showed that the adsorbent had better selectivity for Gd^3+^ and Nd^3+^ than La^3+^ and Y^3+^, indicating that a moderate ion radius was beneficial to adsorption. In another example, Wu et al. [72] developed 3D S-impregnated nano-MnO_2_ nanorods with lengths ranging from 300 to 800 nm for selective removal of Pd^2+^. The competitive adsorption experiment showed that the S atom is a soft base that can bind with a soft acid, Pd^2+^. However, interfering ions Ni^2+^, Cu^2+^, Zn^2+^, and Co^2+^ are hard Lewis acids, so they cannot be adsorbed. Fu et al. [73] also prepared a 2D porous Fe_3_O_4_/Poly(C_3_N_3_S_3_) nanocomposite for selective removal of Pb^2+^ and Hg^2+^; the average size of Fe_3_O_4_ was about 7.5 nm while the pore size of the poly(C_3_N_3_S_3_) network ranged from 5 to 500 nm. The competitive adsorption experiment showed that Hg^2+^ as a soft acid, can be easily bound to S, while Pb^2+^ and Cu^2+^ as intermediate acids have a medium affinity for the nitrogen groups. On the contrary, Mg^2+^ and Ca^2+^ are hard acids, so Fe_3_O_4_/Poly(C_3_N_3_S_3_) has the worst adsorption selectivity for them.

### 3.3. Synergistic Effect

**Figure 10:**
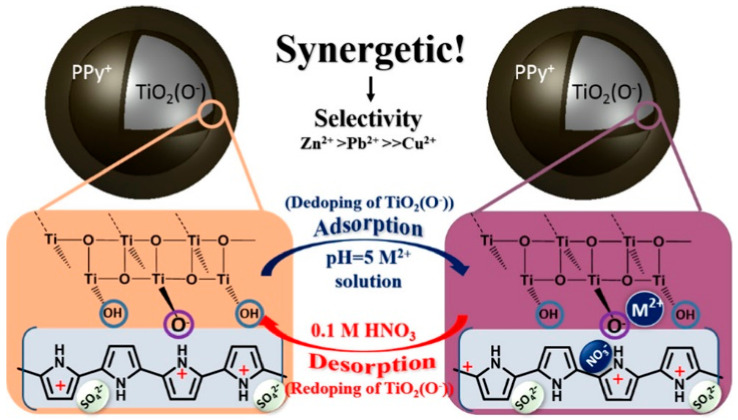
Synergistic adsorption between PPy and TiO_2_ in the PPy/TiO_2_ composite of selective adsorption for heavy metal ions [74].

Different from other adsorption mechanisms, the synergistic effect refers to the combined work of each component, resulting in selective adsorption ability. Chen et al. [74] prepared a PPy/TiO_2_ core-shell composite that can selectively adsorb heavy metal ions. The SEM images showed that TiO_2_ was the core while polypyrrole (PPy) was the shell. It was found that the selective adsorption order was as follows: Zn^2+^ > Pb^2+^ ≫ Cu^2+^, and the result cannot be explained by any known theory. The adsorption mechanism is a synergistic effect, which can be briefly described in Figure 10. PPy can conduct a doping-dedoping process, through which TiO_2_ displays a selective adsorption performance towards Zn^2+^, Pb^2+^, and Cu^2+^. However, the reason for the higher adsorption tendency of TiO_2_ to Zn^2+^ than Cu^2+^ is unknown.

Zhang et al., presented a hydrothermal synthesis of hierarchical rod-like mesoporous Mg-Al bimetallic oxides (Mg/Al-BOs) with a high specific surface area of 472.4 m^2^·g^−1^ and high adsorption capacity, selectivity, and reusability for U(VI) uptake via both surface complexation and electrostatic interaction [75]. The oxygen-containing groups on the surface of Mg/Al-BOs play significant roles in the U(VI) adsorption in addition to the electrostatic attractions, for which the complexation process could be described as follows:

≡ M−O^−^+ H_2_O → ≡ M−OH + OH^−^ (3)

≡ M−OH + U(VI) → ≡M−O−U(VI) + H^+^ (4)

Bian et al. [76] also found a synergistic effect between ferrite and TiO_2_. They synthesized a series of core-shell MFe_2_O_4_-TiO_2_ (M=Mn, Fe, Zn, Co, or Ni) to selectively adsorb toxic UO^2+^ in the presence of Rb^+^, Sr^2+^, Cr^3+^, Mn^2+^, Ni^2+^, Zn^2+,^ and Cd^2+^. The shell MFe_2_O_4_ increased the average diameter of the core TiO_2_ from 2.5 nm to hundreds of nanometers. The adsorption mechanism was embodied as follows: at first, M^2+^ ions act as a mediator to make holes from MFe_2_O_4_ to TiO_2_; then, TiO_2_ on the surface can adsorb all cations with hydroxyl radicals except UO^2+^; after that, the remaining UO^2+^ is adsorbed in the interface of MFe_2_O_4_-TiO_2_ through holes, leading to selective adsorption. Hierarchical Mg-Al bimetallic oxide/straw fiber (Mg-Al/SF) was hydrothermally synthesized and incorporated with the in situ growth method.

Hierarchical Mg-Al bimetallic oxide/straw fber (Mg-Al/SF) was hydrothermally synthesized and incorporated with in the situ growth method. The synergistic combination of surface adsorption from biomass fiber and chemical adsorption from alumina and magnesium oxide made Mg-Al/SF a promising adsorbent for enhanced phosphorus removal with the maximum adsorption capacity of 89.37 mg/g [77].

### 3.4. Special Functionalization

Surface functionalization is an efficient method to reduce the agglomeration of the powder adsorbents, and it can also improve their stability and adsorption performance. Some selective adsorptions are achieved by special functionalized adsorbents aimed at the adsorbate, such as specific ion exchange, special substances, and specific chemical bonds. Kera et al. [78] prepared a PPy-PANI/Fe_3_O_4_ core-shell nanocomposite with mean sizes of 50–100 nm for selective adsorption of Cr(VI). The protonated N atoms on the surface of PPy-PANI/Fe_3_O_4_ can adsorb Cr(VI) in acidic conditions, and realize selective ion exchange of Cr(VI) with innocuous Cl^−^. Therefore, neither cation nor anion could affect the adsorption, and the maximum adsorption capacity can reach 434.78 mg·g^−1^. Futher, Fe_3_O_4_ nanoparticles were functionalized by a modified mussel-inspired method with dopamine (DA) and (3-aminopropyl)triethoxysilane (KH550), thus obtaining core-shell Fe_3_O_4_/poly(DA+KH550) hybrids for MB. The surface of the hybrid is negatively charged at a higher pH, resulting in interaction with MB molecules via electrostatic attraction, hydrogen bonding, and π–π stacking, and it showed a maximum adsorption capacity of 400.00 mg·g^−1^ for MB, easy separation, and excellent reusability characteristics because of the high specific surface area and abundant active adsorption sites [79].

Similarly, Wu et al. [80] prepared a La(OH)_3_/Fe_3_O_4_ core-shell nanocomposite with a maximum adsorption capacity of 83.5 mg·g^−1^ for phosphate, and the adsorption process was described as follows:

La − OH + H_2_PO_4_^−^ ↔ La − H_2_PO_4_ + OH^−^ (5)

La = OH + HPO_4_^2−^ ↔ La = HPO_4_ + 2OH^−^ (6)

La ≡ OH + PO_4_^3−^ ↔ La ≡ PO_4_ + 3OH^−^ (7)

It seems that harmless –OH groups on La can selectively exchange phosphorus groups in the actual wastewater containing ions of various concentrations. This result was the same as the previous research about phosphate removal via La-based materials, including lanthanum oxides or lanthanum hydroxides [81].

Some substances or groups have a specific adsorption affinity for certain toxic metals. For example, Cs^+^, a widely present rare metal element ion in nuclear radiation wastewater, can be specifically adsorbed by Prussian Blue (PB), which is analogous because it has specific recognition ability for Cs^+^ [82,83]. PB can form specific lattices with Cs^+^ because its lattice parameter and hydrated ion radius of ^137^Cs^+^ are well matched. U(VI), similar to Cs^+^, can preferentially form complexes with phosphorous groups [35,36,84]. As for fluorine-containing pollutants, it is a good choice to choose fluorine-containing functional groups to construct adsorbents due to the fluorine-fluorine (F-F) interaction [85,86]. Table 2 summarizes the advantages and disadvantages of some current adsorption selection mechanisms. It was found that adsorbents with good performance can be prepared by combining the properties of the adsorbates and materials to be used.

## 4. Conclusions

This review mainly focuses on the synthesis methodologies of hierarchical oxide composites, their selective adsorption performance, and adsorption mechanisms for hazardous substances from the aqueous matrix. Their greatest advantage for the removal of pollutants is that they can combine the advantages of the physico-chemical characteristics of all the components and their multi-level structures with diverse morphologies that realize the effect of “1 + 1 > 2”. However, there still exist some problems for most of the functionalized hierarchical oxides that influence their selective adsorption performance, such as the agglomeration of magnetic Fe_3_O_4_ modified by polymers. Future research should focus on retaining their original hierarchical morphology with good dispersibility. Secondly, although many studies have reported their applications for removing various pollutants, including heavy metal ions, organic dyes, etc. Most of them are not suitable for mass-scale preparation for large-scale applications. Therefore, it is of increasing interest to explore high-efficiency methods for controllable-scale fabrication of hierarchical oxide composites. Thirdly, though surface functionalization has been proven to be an effective method to improve the adsorption performance of hierarchical oxides, the amounts of their functional groups for binding pollutants are strongly dependent on the functionalization method, whereas the functionalization process of most reported functionalized adsorbents is cumbersome and time-consuming. Fourthly, simple wet chemical routes, including hydrothermal/solvothermal methods, coprecipitation methods, and sol-gel methods are largely limited in utility due to the low pore density of agglomeration and hence the low specific surface area. To simultaneously avoid these shortcomings, offer monodispersed structures, and achieve high functionality, soft templates and self-assembled composites need to be further explored in the near future. Fifthly, most studies are static adsorption for the specific model pollutants prepared in the laboratory, and they almost have no practical application potential. Finally, it is quite difficult for the hierarchical oxide composites to in-situ monitor their adsorption processes for pollutants, and thus their thorough removal mechanisms for pollutants are unclear in detail. Therefore, future studies should pay attention to the in-situ adsorption process and the dynamic selective adsorption of the hierarchical oxide composites for actual industrial wastewater. In short, this review contains systematically gathered information using highly related references on selective adsorption of hazardous substances from wastewater by hierarchical oxide composites, and thus can serve as a starting point for research in the related field.

## Figures and Tables

**Table 1:** Comparison of different methods for the preparation of hierarchical oxides.

Methods	Advantages	Disadvantages
Hydrothermal/solvothermal method	Beneficial to crystal nucleation and growth, easy to control morphology.	Slight aggregation, not easy to control in a closed space; relatively long reaction time; high reaction temperature; and high pressure.
Coprecipitation	Usually involving metal salts and precipitants, mild reaction conditions usually occur at room temperature.	Only be used to prepare metal oxides; similar precipitation equilibrium constants of metal salts.
Template	Precise control of the size, morphology, and structure, aggregation reduction of nanoparticles.	Structure destruction while removing the template, limits of the reaction conditions for the template.
Polymerization	Combination of polymer and metal oxide to form composites with more functional groups.	Complex preparation process, easy aggregation resulting in destroyed morphology.
Sol-gel	Easy separation, beneficial to form a 3D network, and homogeneous mixing of components at the molecular level.	Long reaction time for several days, making it easy to introduce anionic impurities.

**Table 2:** Comparison of different selective mechanisms.

Mechanism	Advantage	Disadvantage	Selectivity
Molecularly imprinted polymer or ion imprinting technology	Adsorption of a specific adsorbate by introducing a corresponding template, not easily affected by external factors	Complex preparation method, only adsorption for single adsorbate, bad morphology	Excellent
Surface charge effect	Adsorption of many adsorbates with the same charge, wide scope of application	Affected by pH value, influenced by coexisting ions with the same charge	Good
Hard-soft-acid-base theory	Suitable for atomically modified adsorbents	Limited scope of application, influenced by amphoteric coexisting ions	Good
Synergistic effect	No modification results in good morphology; the simple preparation of adsorbent	Speculative and unclear adsorption mechanism, limited scope of application	Good
Special functionalization	Adsorption of a specific adsorbate, not affected by external factors	Useless to multiple pollutants, limited scope of application	Excellent

## Data Availability

The data underlying this article are available in the corresponding references.
